# Phagocytosis depends on TRPV2-mediated calcium influx and requires TRPV2 in lipids rafts: alteration in macrophages from patients with cystic fibrosis

**DOI:** 10.1038/s41598-018-22558-5

**Published:** 2018-03-09

**Authors:** Manuella Lévêque, Aubin Penna, Sophie Le Trionnaire, Chantal Belleguic, Benoît Desrues, Graziella Brinchault, Stéphane Jouneau, Dominique Lagadic-Gossmann, Corinne Martin-Chouly

**Affiliations:** 1 Research Institute for Environmental and Occupational Health (IRSET) INSERM U1085, team ‘Stress, Membrane and Signaling, F-35043 Rennes, France; 2 University of Rennes 1, UMS Biosit, F-35000 Rennes, France; 3 Centre Hospitalier Universitaire de Rennes, Centre de Ressource et de Compétences de la Mucoviscidose, F-35064 Rennes, France; 4 Research Institute for Environmental and Occupational Health (IRSET) INSERM U1085, team ‘Chemical contaminant immunity and inflammation, F-35043 Rennes, France; 5 URL440-COSS, Centre Eugène Marquis, F-35064 Rennes, France

## Abstract

Whereas many phagocytosis steps involve ionic fluxes, the underlying ion channels remain poorly defined. As reported in mice, the calcium conducting TRPV2 channel impacts the phagocytic process. Macrophage phagocytosis is critical for defense against pathogens. In cystic fibrosis (CF), macrophages have lost their capacity to act as suppressor cells and thus play a significant role in the initiating stages leading to chronic inflammation/infection. In a previous study, we demonstrated that impaired function of CF macrophages is due to a deficient phagocytosis. The aim of the present study was to investigate TRPV2 role in the phagocytosis capacity of healthy primary human macrophage by studying its activity, its membrane localization and its recruitment in lipid rafts. In primary human macrophages, we showed that *P*. *aeruginosa* recruits TRPV2 channels at the cell surface and induced a calcium influx required for bacterial phagocytosis. We presently demonstrate that to be functional and play a role in phagocytosis, TRPV2 might require a preferential localization in lipid rafts. Furthermore, CF macrophage displays a perturbed calcium homeostasis due to a defect in TRPV2. In this context, deregulated TRPV2-signaling in CF macrophages could explain their defective phagocytosis capacity that contribute to the maintenance of chronic infection.

## Introduction

Phagocytosis of bacteria by macrophage is a complex, multistep physiological process critical for defense against invading pathogens and hence for innate immunity. The common unfolding of phagocytosis includes pathogens recognition by specific receptors, actin cytoskeleton rearrangement, and protein clustering leading to particle internalization^1^. Calcium and sodium ions play an important role in the different steps of phagocytosis including phagolysosomes acidification^2^. A localized cytosolic Ca^2+^ gradients is required in particular to generate the signals necessary for Fcγ receptors mediated phagocytosis^3–6^.

Among calcium and sodium channels, TRPV2 (transient receptor potential vanilloid 2) was shown to have a pivotal role in macrophage particle binding and phagocytosis in mice^7,8^. TRPV2 is a nonselective cation channel that can be activated by many stimuli including heat, insulin, cannabinoids and phosphatidylinositol-3-OH kinase (PI3K) signaling^9^. It may hold a role in regulating basal calcium homeostasis. TRPV2 is abundantly and ubiquitously expressed in cells from the innate immune system including macrophages. In murine macrophages, TRPV2 was demonstrated to participate in the early phagocytosis in response to zymosan-, immunoglobulin G (IgG) and complement-mediated particle binding and phagocytosis. TRPV2 involvement in the phagocytosis processes included PI3K dependent recruitment of TRPV2 at the plasma membrane leading to its activation and a subsequent depolarization of the plasma membrane ending to actin cytoskeleton reorganization^7,10^.

Localization of receptors or channels at the plasma membrane is critical for signaling and phagocytosis processes. Notably, recruitment of certain TRP channels in lipid rafts modulates their activity^11,12^. Lipid rafts are lipid microdomains enriched in sphingolipids and cholesterol that exhibit functions in membrane signaling and trafficking. They exist as distinct liquid-ordered phases of the membrane. The composition and biophysical properties of plasma membrane may play a crucial role in phagocytosis processes. Actually, upon stimulation of macrophages, the plasma membrane undergoes condensation to form highly ordered phagosomal membranes, a biophysical hallmark of lipid raft^13^.

Cystic fibrosis (CF) is an autosomal recessive disorder caused by mutations in the gene encoding for the Cystic Fibrosis Transmembrane Conductance Regulator (CFTR) protein; it is the most common genetic disorder affecting the Caucasian population. CF is characterized by multiple infections due to an alteration of the mucociliary clearance^14^ and an impaired capacity of macrophages to phagocyte and kill bacteria^15–19^. Moreover, infection by *Pseudomonas aeruginosa* (*P*. *aeruginosa*) increases in adults with CF leading to chronic infection^20^, loss of lung function, supporting a bad prognosis and increasing morbidity and mortality of patients with CF. Nevertheless, the mechanisms responsible for the alteration of CF macrophage phagocytosis capacity is not clearly understood, particularly during the first step when the phagocytic cup is formed. This study aimed at seeking these mechanisms by looking at membrane properties and the potential role of TRPV2. We demonstrate here that in human macrophage, bacterial infection recruits TRPV2 channels at the plasma membrane, particularly in lipid rafts, and triggers a TRPV2-like calcium signal required for phagocytosis. Moreover, macrophages from CF patients display altered TRPV2 expression and activity. Impaired TRPV2 function in CF macrophages might be responsible of their inability to phagocyte bacteria.

## Results

### Bacterial phagocytosis by primary human macrophages depends on a TRPV2-like calcium signal

**Figure 1:**
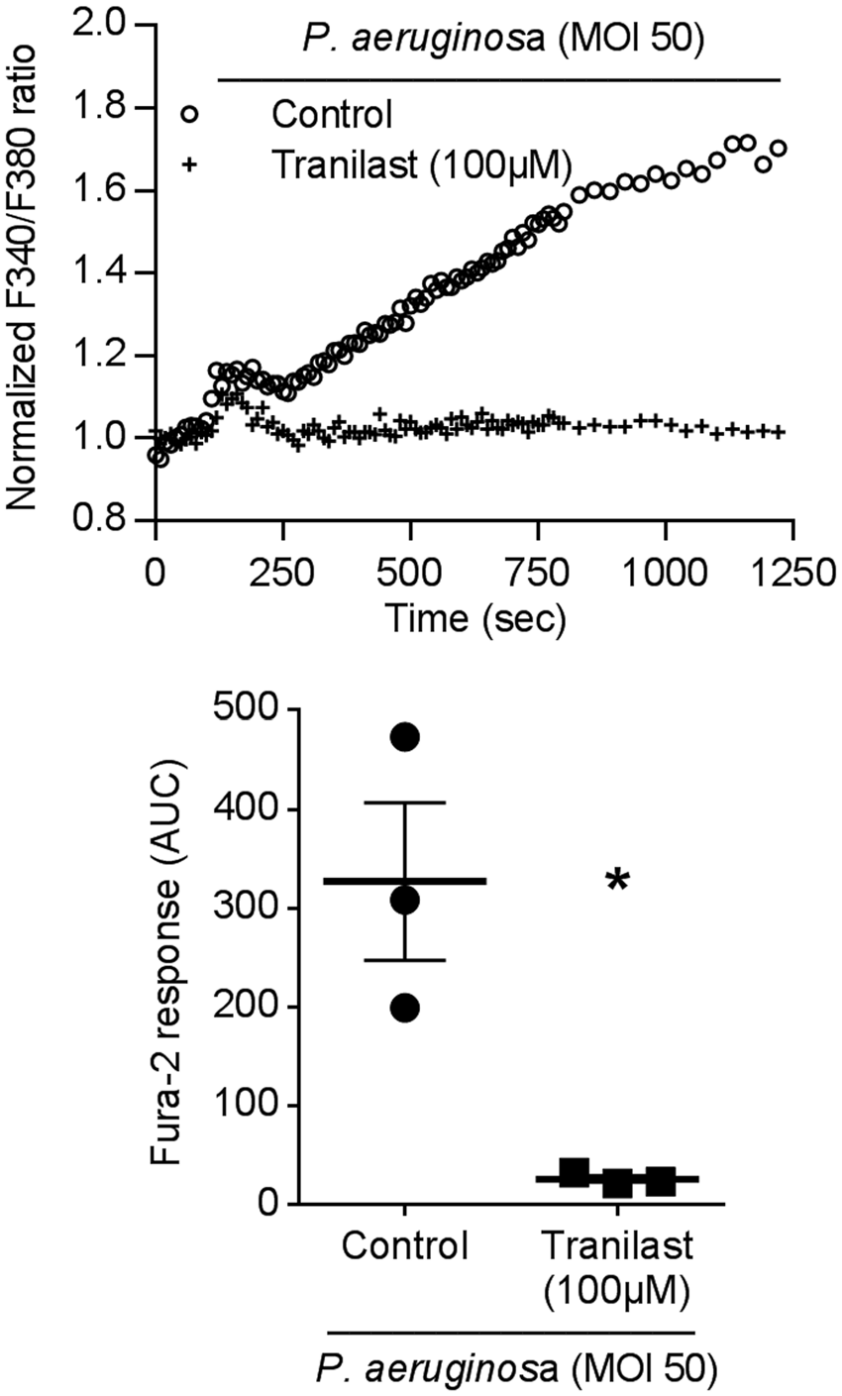
*P*. *aeruginosa* induced a TRPV2-mediated calcium influx in primary human macrophage. TRPV2 mediated-Ca^2+^ influx was measured in macrophages stimulated by *P*.*aeruginosa* (MOI 50) in the absence (control) or presence of tranilast (100 µM, 15 min preincubation). Data are depicted as the ratio of emission after excitation at 340 nm relative to that after excitation at 380 nm (F340/F380) and normalized to basal level 1. Horizontal bars represent stimulus period. Data are representative of three independent experiments. Below, area under the curve of similar experiments are shown as mean ± s.e.m. Mann-Whitney test: *p < 0.05 *vs*. control.

**Figure 2:**
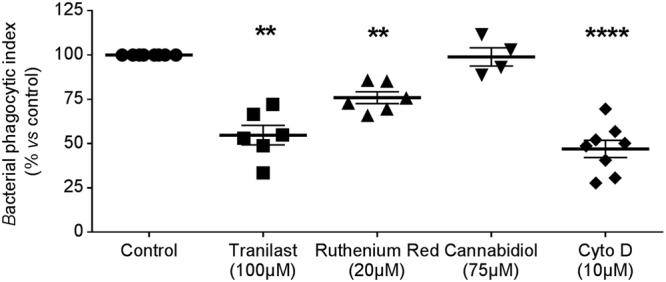
Phagocytosis is dependent on TRPV2 activation. Phagocytosis capacity was evaluated using heat-inactivated *E*. *coli* bound to fluorescein. Bacterial phagocytic index was determined under control condition or after treatment with tranilast (100 µM, n = 6), ruthenium red (20 µM, n = 6) and cannabidiol (75 µM, n = 4). Cytochalasin D (Cyto D, 10 µM) is used as negative control (n = 7). Data are shown as mean ± s.e.m. Each experiment was realized in quadruplicat. Mann-Whitney test: **p < 0.01 and ****p < 0.0001 *vs*. control.

TRPV2 is expressed in blood-derived monocytes and *in vitro* differentiated macrophages (Data not shown). To test the role of TRPV2 in human macrophage phagocytosis, we have first investigated the impact of bacterial infection on intracellular calcium homeostasis. In primary human macrophages, infection with *P*. *aeruginosa* induced a biphasic calcium signal (Fig. 1). Interestingly, the larger sustained calcium increase is blocked by tranilast, the most specific TRPV2 pharmacological inhibitor actually known^9,21–23^. Similar results were obtained after heat-inactivated *E*. *coli* infection (Supplementary Fig. S1). These results suggest that *P*. *aeruginosa-*induced calcium influx is at least partly dependent on TRPV2 activity (Fig. 1). Next, we aimed at determining whether this TRPV2-like calcium signal is required for the bacterial phagocytosis process. To do so, phagocytosis assays were performed in human primary macrophages, with or without tranilast or ruthenium red, a broad-spectrum antagonist of TRPV channels. Both compounds significantly decreased macrophages phagocytosis capacity with a stronger effect for tranilast, making the bacterial phagocytic index dropped to a level similar to the one observed with cytochalasin D used as positive control (Fig. 2). Cannabidiol, a TRPV2 channel activator, did not have any effect on phagocytosis (Fig. 2). These results showed that efficient bacteria phagocytosis by human macrophage seems to require TRPV2 activity.

### *P*. *aeruginosa* infection triggers TRPV2 recruitment at the plasma membrane, especially in lipid rafts

**Figure 3:**
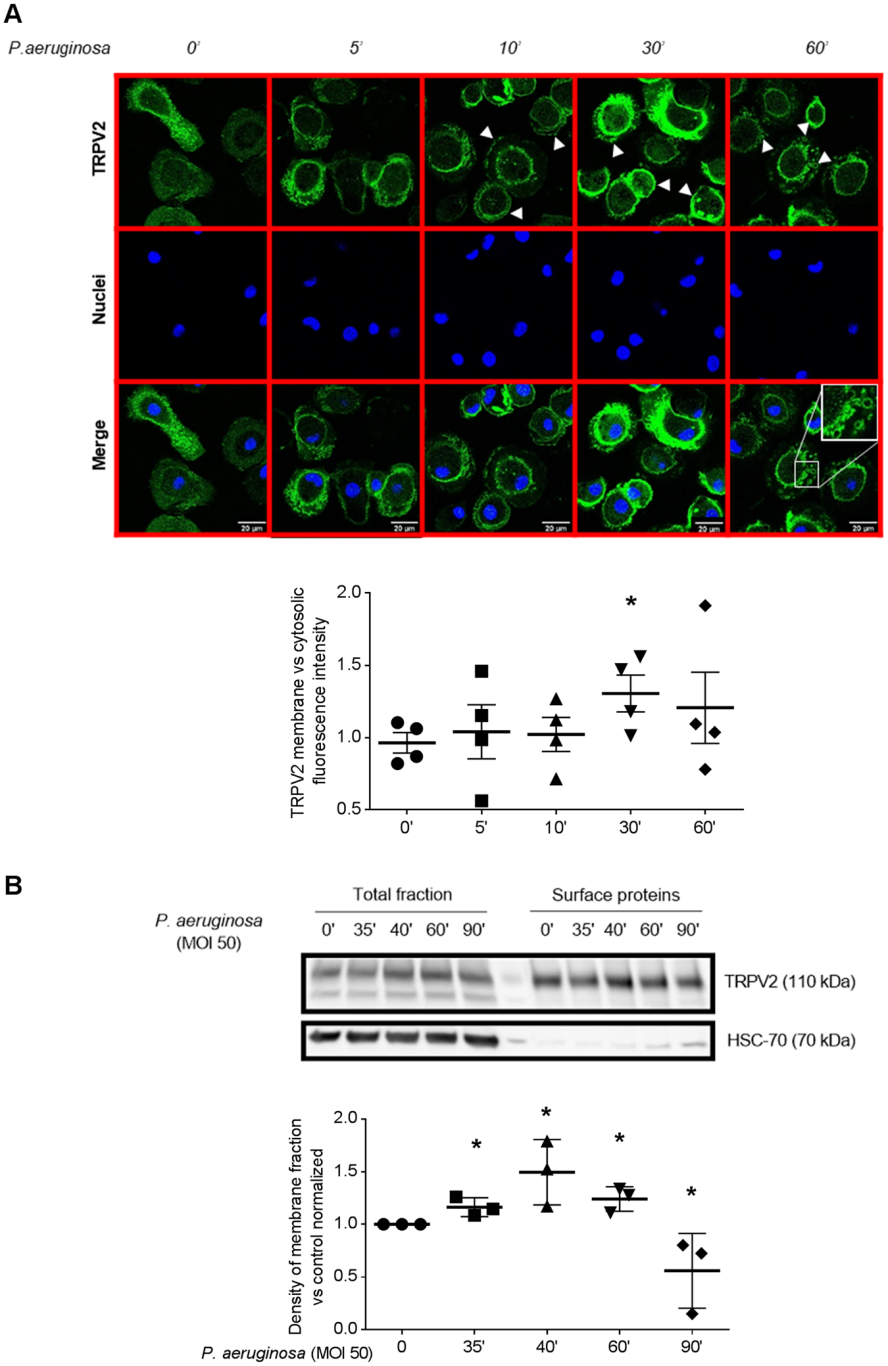
*P*. *aeruginosa*-induced TRPV2 recruitment to plasma membrane. (**A**) Confocal pictures of TRPV2 localization in human primary macrophages after *P*. *aeruginosa* treatment (MOI 50, time 0 to 60 min). Images are representative of four separate experiments. Data representative of the TRPV2 surface relative to cytosolic fluorescence are shown below as mean ± s.e.m. Each point are representative of the fluorescence quantification of an average of 32 cells totally observed in at least three fields. Mann-Whitney test: *p < 0.05 *vs*. control. (**B**) Surface proteins biotinylation experiments in human macrophages treated with *P*. *aeruginosa* (MOI 50, time 35 to 90 min). Total cellular lysates and the biotinylated cell surface fractions were resolved on reducing gel and analyzed by immunoblotting. HSC-70 is used as control of membrane integrity. Representative blot of four separate experiments are shown. Densitometric analysis of TRPV2 surface expression are shown as mean ± s.e.m. Full-length blots are presented in Supplementary Figure 4. The results were expressed *vs*. the relative intensity observed at 0 min and normalized by total fraction. Mann-Whitney test: *p < 0.05 *vs*. control.

Several evidences exist showing that TRPV2 channels are constitutively active and are regulated by a dynamic trafficking to cell surface^7,10^. To further confirm that TRPV2 activity is recruited during bacterial infection and contributes to phagocytosis, we investigated TRPV2 subcellular localization in response to *P*. *aeruginosa*. Confocal pictures analysis of cells infected with *P*. *aeruginosa* for 5, 10, 30 or 60 min showed an increase of TRPV2 level at the plasma membrane from 30 min, and a reduction at 60 min. Moreover, doughnut-shaped intracellular TRPV2 staining could be detected at 60 min and could correspond to internalized bacteria-containing phagosomes (Fig. 3A). TRPV2 membrane translocation was further confirmed using surface protein biotinylation assay. Macrophage membrane surface proteins were biotinylated (30 min) after *P*. *aeruginosa* infection (5 to 60 min), isolated and analyzed for TRPV2 level by immunoblotting compared to total TRPV2 expression. As expected, after 10 min infection with *P*. *aeruginosa* and 30 min biotinylation, a significant increase of the presence of TRPV2 at plasma membrane was observed (Fig. 3B). These results suggest that bacterial infection recruits TRPV2, at least in part by triggering TRPV2 plasma membrane targeting.

**Figure 4:**
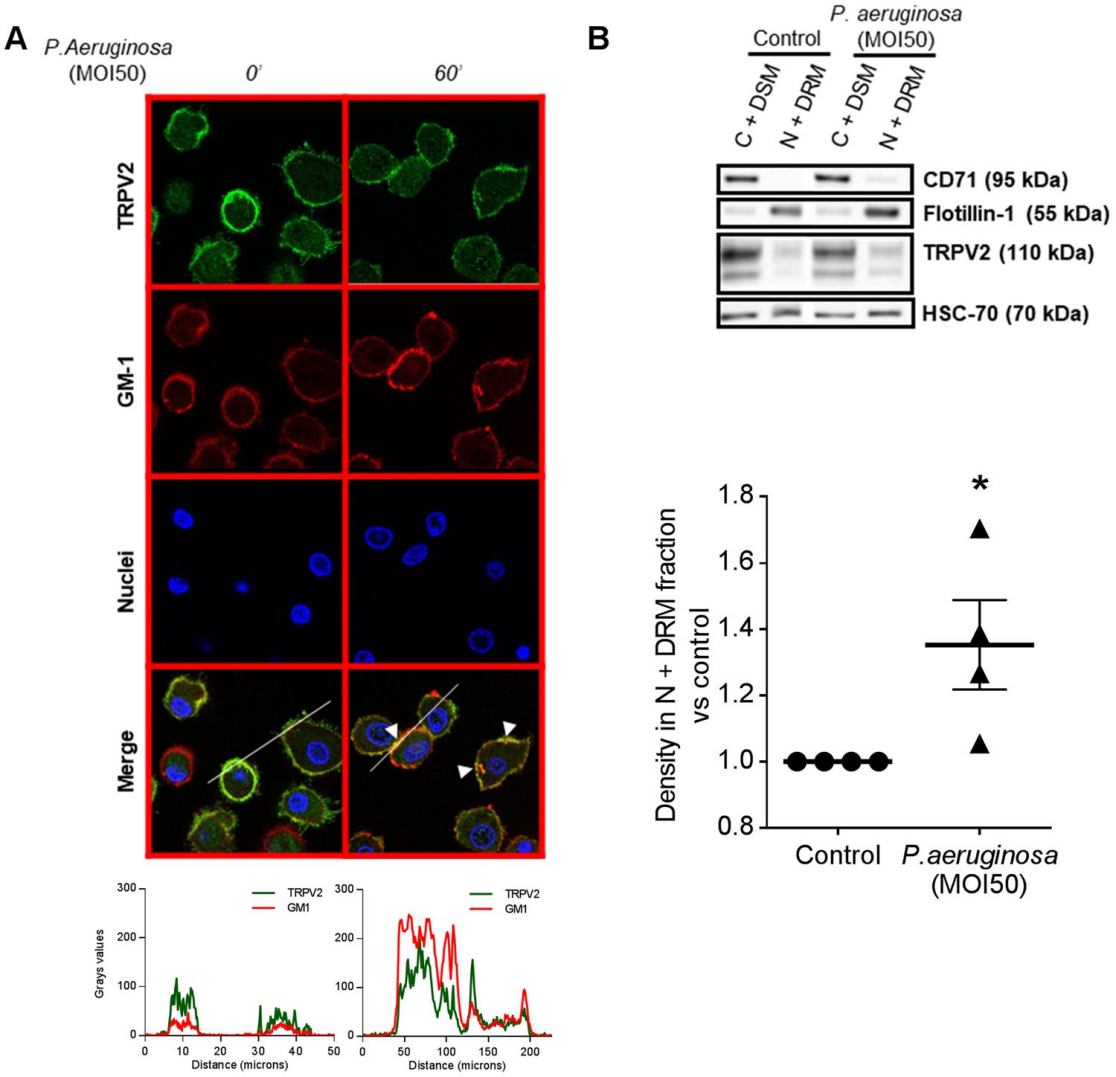
*P*. *aeruginosa*-induced TRPV2 recruitment into lipid rafts at the plasma membrane of macrophages. (**A**) Confocal images of TRPV2 localization in human primary macrophages after *P*. *aeruginosa* treatment (MOI 50, 60 min). Colocalization of TRPV2 staining with the raft marker dye, CTX-B, which recognizes the GM1 ganglioside. Data are representative of three independent experiments corresponding to an average of 63 cells from at least four fields each. Fluorescence profiles corresponding to the line are present below. (**B**) Human macrophages infected with *P*. *aeruginosa* (MOI 50, 60 min) were analyzed by quantitative separation of C + DSM (cytoplasm and detergent-soluble membrane) and N + DRM (nuclei and detergent-resistant membranes) fractions using lysis gradient centrifugation. Both fractions were resolved on reducing gel and analyzed by immunoblotting. Representative blots of four separate experiments are shown. CD71 and flotillin-1 are cell compartment markers for C + DSM and N + DRM respectively. In the histogram, the densitometric analysis of TRPV2 in the N + DRM fraction of the same experiments are shown as mean ± s.e.m. Full-length blots are presented in Supplementary Figure 4. The results were expressed *vs*. the relative intensity observed in control. Mann-Whitney test: *p < 0.05 *vs*. control.

Because lipid rafts are membrane platforms important for both the phagocytic function and TRP channel activity, we wondered whether lipid raft localization might regulate TRPV2 activity. First, TRPV2 presence in lipid rafts was investigated by confocal imaging using a lipid raft marker, the monosialotetrahexosyl ganglioside GM1, detected with Alexa fluor 647-labelled cholera toxin-B (CTX-B) that binds the pentasaccharide chain. After 60 min of *P*. *aeruginosa* infection, corresponding to a time window between TRPV2 recruitment at the plasma membrane and phagosome maturation, clusters of co-localized TRPV2 and GM1 ganglioside were observed, suggesting that TRPV2 lipid raft targeting could be important in the phagocytosis processes of *P*. *aeruginosa* (Fig. 4A). Next, using iodixanol gradients, we isolated lipid raft from the rest of the membrane before and after 60 min of *P*. *aeruginosa* infection and analyzed the amount of TRPV2 present in each fraction by immunoblotting (Fig. 4B). In resting cells, TRPV2 is almost exclusively present in non-raft membranes (C + DSM, cytoplasm and detergent-soluble membrane) while bacterial infection increase significantly the level of TRPV2 detected in the enriched lipid rafts fraction (N + DRM, nuclei and detergent-resistant membranes). Treatment of macrophages with LPS from *P*. *aeruginosa* gave the same results (Supplementary Fig. S2A) further supporting the fact that TRPV2 is recruited in lipid rafts during infection (Fig. 4B). Note that the fractionation procedure and the lipid raft (N + DRM) fraction purity were controlled using the lipid raft marker flottillin-1 and the non-raft marker CD71 (Fig. 4B), as well as for the enrichment in cholesterol content (Supplementary Fig. S2B).

**Figure 5:**
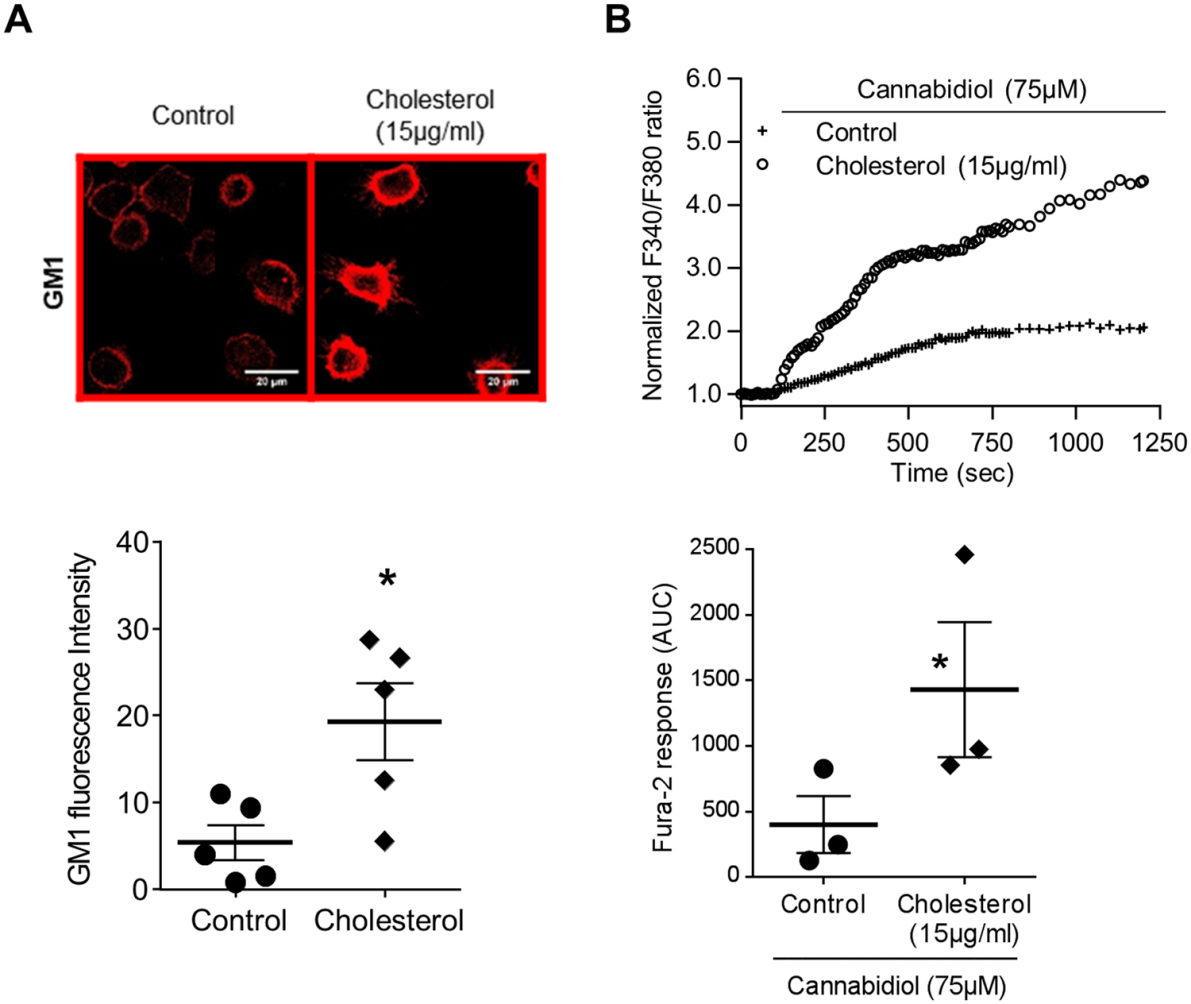
Addition of exogenous cholesterol increased cannabidiol-induced TRPV2 calcium influx. (**A**) Confocal images with the raft marker dye, CTX-B, which recognize the GM1 ganglioside localization in human primary macrophages after cholesterol treatment (15 µg/ml). Images are representative of five separate experiments. Data representative of the GM1 fluorescence are shown below as mean ± s.e.m. Each point are representative of the fluorescence quantification of at least 30 cells coming from at least five fields. Mann-Whitney test: *p < 0.05 *vs*. control. (**B**) Fura-2 AM calcium measurement was performed in human macrophages stimulated by cannabidiol (75 µM) in the absence (control) or presence of cholesterol (15 µg/ml, 30 min pretreatment). Data are presented as the ratio of emission after excitation at 340 nm relative to that after excitation at 380 nm (F340/F380) and normalized to basal level 1. Horizontal bar represented stimulus period. Data are representative of three independent experiments. Below, area under the curve of similar experiments are shown as mean ± s.e.m. Mann Whitney test: *p < 0.05 *vs*. control.

Finally, in order to probe the impact of TRPV2 lipid-raft localization on TRPV2-mediated calcium influx, we evaluated the effect of water-soluble cholesterol. This treatment increases plasma membrane cholesterol level and the size of the lipid rafts domains leading to rigid zones in the plasma membrane (Fig. 5A). Under such conditions, a significant amplification of the TRPV2-mediated calcium signal was detected (Fig. 5B). Taken together, these results demonstrated that rigid zone formation at the plasma membrane potentiate TRPV2 activity and showed the importance of TRPV2 recruitment in lipid rafts to mediate calcium influx upon infection.

### Impaired TRPV2 expression and TRPV2 activity in human CF macrophages

**Figure 6:**
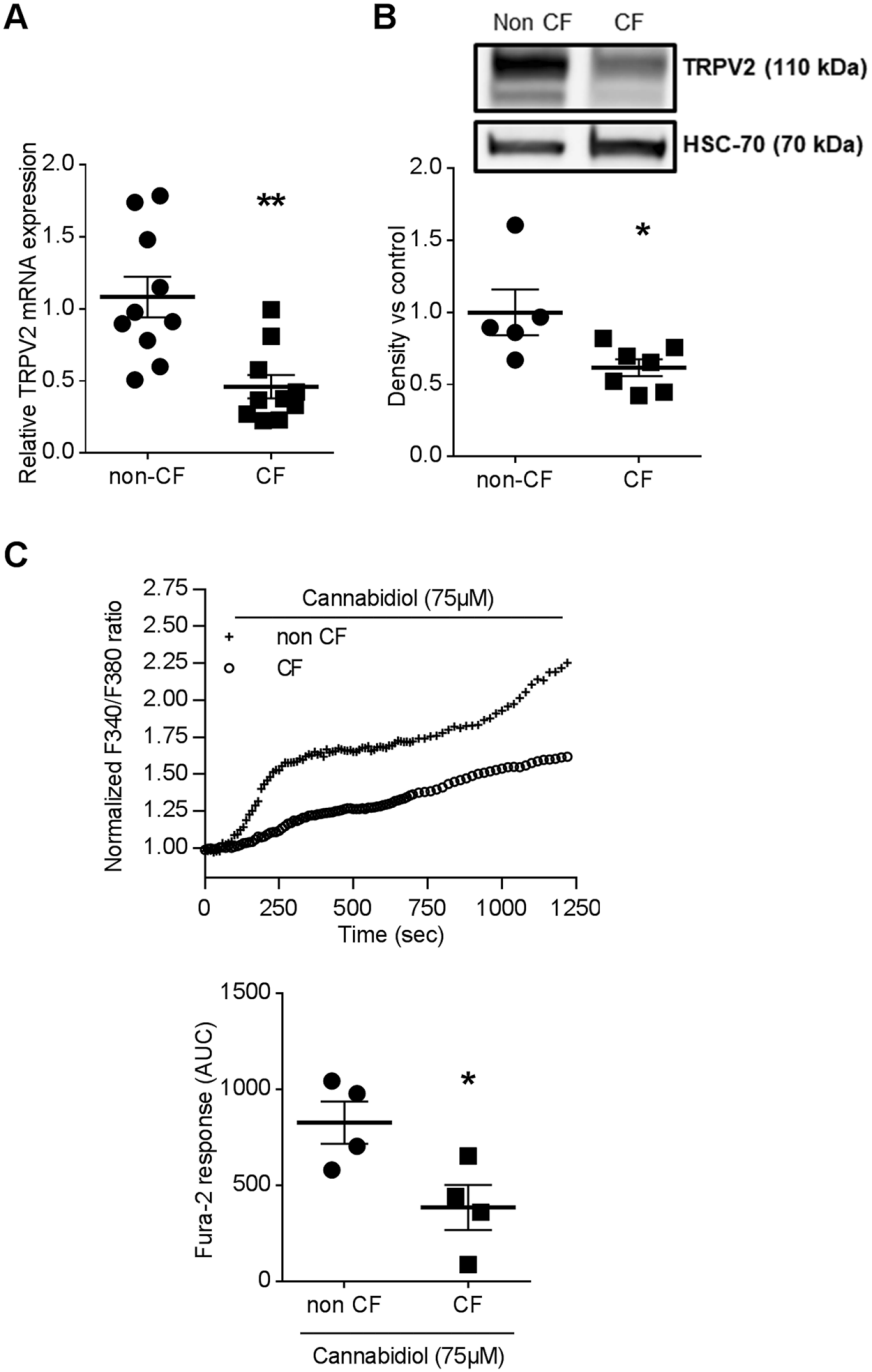
Defective TRPV2 expression and activity in human CF macrophages. (**A**) TRPV2 gene expression in non-CF (n = 10) and CF macrophages (n = 10, patients 1–10, supplementary Table S1). TRPV2 mRNA expression were determined by RT-qPCR. Mann-Whitney test: **p < 0.01 *vs*. non-CF macrophages. (**B**) In a representative blot, TRPV2 (110 kDa) expression is observed on total cellular lysate from non-CF (n = 5) and CF (n = 8, patients 11–18, supplementary Table S1) macrophages. Equal protein loading was controlled *via* HSC70 detection. Full-length blots are presented in Supplementary Figure 4. The scatter dot plot represents densitometric analysis. Results are shown as the mean ± s.e.m. Mann-Whitney test: *p < 0.05 vs. non-CF macrophages. (**C**) Fura-2 AM calcium measurement in non-CF and CF macrophages stimulated by cannabidiol (75 µM) (n = 4, patients 19–22, supplementary Table S1). Data are presented as the ratio of emission after excitation at 340 nm relative to that after excitation at 380 nm (F340/F380), and normalized to basal level 1 and to maximal level 4 (ionomycin). Horizontal bar represented stimulus period. Below, area under the curve of similar experiments are shown as mean ± s.e.m. Mann-Whitney test: *p < 0.05 *vs*. non-CF macrophages.

We have previously demonstrated that human CF macrophage exhibits an altered phagocytosis capacity^18^. To evaluate whether this defect could be explained by a deregulation of TRPV2 signaling, we quantified TRPV2 expression levels in healthy (non-CF) and CF human macrophages. Interestingly, we observed that both TRPV2 gene and protein expression are significantly decreased in CF *vs* non-CF macrophages (Fig. 6A,B). In addition, cannabidiol, a known TRPV2 activator, induced smaller calcium responses in human CF macrophages compared to non-CF cells (Fig. 6C). Cannabidiol is relatively selective for TRPV2 at the concentration used but it has been shown that this compound can modulate other channels activities, especially TRPM8, TRPA1 and TRPV1. In human monocyte-derived macrophage TRPM8 and TRPA1 are not expressed (means of Ct are 33.32 and 34.19 respectively, n = 12). TRPV1 are expressed but their expression is not changed in CF macrophages and this channel is not involved in phagocytosis (Supplementary Fig. S3A). Finally, TRPV4 expression is lesser in CF *vs* non-CF macrophages (Supplementary Fig. S3B) but cannabidiol effects on this channel are more controversial. Thus, while we cannot totally rule out potential off-target effects of cannabidiol on channels other than TRP channels, our results strongly suggest that cannabidiol triggers mainly TRPV2 activity in primary human macrophages and that TRPV2 signaling is impaired in human primary CF macrophages.

**Figure 7:**
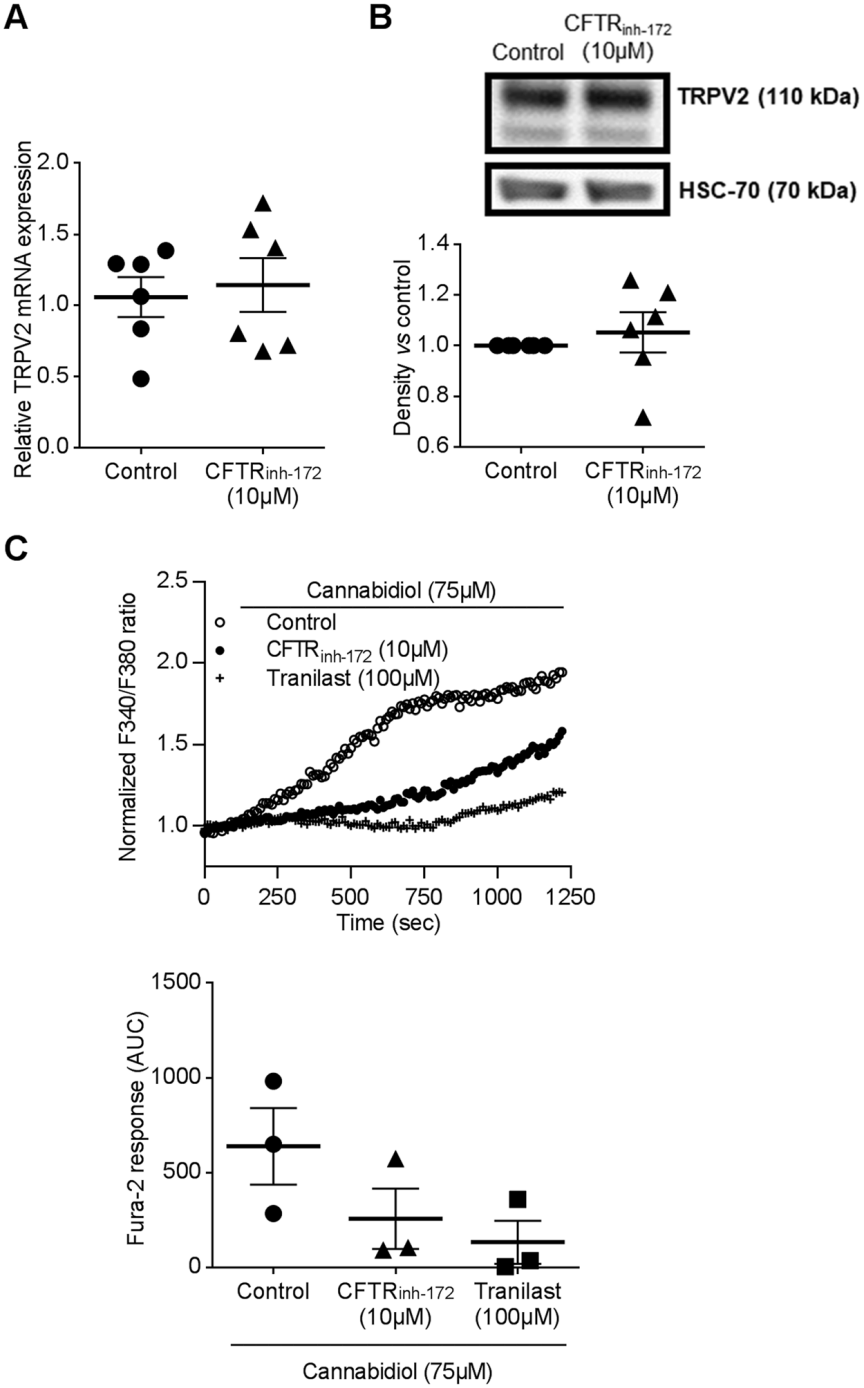
CFTR inhibition decreased TRPV2 mediated-Ca^2+^ influx. (**A**) TRPV2 gene expression in macrophages treated or not with CFTR_inh-172_ (10 µM, 72 h pretreatment) (n = 6). TRPV2 mRNA expression was determined by RT-qPCR. (**B**) Representative blot of TRPV2 (110 kDa) protein level in total cellular lysate from human macrophage in the absence (control) or presence of CFTR_inh-172_ (10 µM, 72 h pretreatment). Equal protein loading was controlled via HSC70 detection. Immunoblots are representative of four independent experiments. Full-length blots are presented in Supplementary Figure 3. The scatter dot plot represents densitometric analysis. Results are shown as the mean ± s.e.m. (**C**) Fura-2 AM calcium measurement in human macrophages cells stimulated by cannabidiol (75 µM) in the absence (control) or presence of CFTR_inh-172_ (10 µM, 72 h pretreatment). Tranilast (100 µM) is used as control for inhibition of TRPV2 mediated-Ca^2+^ influx. Data are given as the ratio of emission after excitation at 340 nm relative to that after excitation at 380 nm (F340/F380), and normalized to basal level 1. Data are representative of three independent experiments. Below, area under the curve of similar experiments are shown as mean ± s.e.m. Mann Whitney test.

We have shown that alteration of CF macrophage phagocytosis capacity is linked to CFTR malfunction^18^. In order to test a possible direct cross-talk between TRPV2-dependent calcium entry and CFTR ion transport activity, we pharmacologically inhibited CFTR function in non-CF macrophages by CFTR_inh-172_ (10 µM, 72 h), often used at this concentration in that context^17,18,26–28^. Interestingly, while CFTR inhibition did not affect TRPV2 gene and protein expression in total lysates (Fig. 7A,B), it decreased cannabidiol-induced TRPV2 calcium influx (Fig. 7C) that could reveal a direct functional interaction between CFTR and TRPV2 activities. However, as inhibition of CFTR function only influences TRPV2 activity under these experimental conditions, alternative mechanisms or signaling pathways are probably involved in the reduction of TRPV2 expression observed in CF macrophages.

Nevertheless, these results evidenced a remodeling of both TRPV2 expression and activity in CF macrophages that might explain why CF macrophage have impaired phagocytosis capacity.

## Discussion

Macrophages are antigen-presenting phagocytes that secrete pro-inflammatory mediators and antimicrobial factors when challenged by extracellular pathogens. They allow bacterial clearance by recognition, phagocytosis and killing.

In recent years, a number of reports have shown the importance of calcium signaling in these functions and have provided evidences of the expression of several members of the TRP channels superfamily in monocytes/macrophages. Among all members of the TRPV family, Nagasawa *et al*. found that only TRPV2 is expressed in macrophages. TRPV2 is a non-selective cation channel showing Ca^2+^ permeability^29^.Using the mouse macrophage cell line TtT/M87 and an EGFP-tagged TRPV2 construct, these authors showed that TRPV2 located in ER rapidly trafficked to the plasma membrane in response to serum or fMLP^10^. fMLP-induced TRPV2-mediated Ca^2+^ influx is required for fMLP-induced macrophage migration. In more recent work, the same laboratory reported that fMLP promoted localization of TRPV2 to podosomes with concomitant increase in subplasmalemmal Ca^2+^ concentration^30^. In addition, TRPV2 was found to be expressed both in plasma membrane and early endosome, where it may serve as an endosomal calcium release channel that controls endosome fusion and/or exocytosis^31,32^. Reinforcing TRPV2 involvement in macrophage functions, Link *et al*. showed that TRPV2-deficient mouse macrophages exhibit impaired *in vitro* phagocytic activity, as manifested by poor phagocytosis in response to zymosan, immunoglobulin G or complement; this was in line with the observation that TRPV2-deficient mice were notoriously sensitive to *Listeria monocytogenes* infection^7^. Cannabidiol is so far the best pharmacological agonist of TRPV2 described^9^ and was identified as TRPV2 agonist using cell-based calcium mobilization and electrophysiological assays^24,25^. But this compound is also known to activate/inhibit other TRP channels (e.g. TRPV1, TRPV4, TRPA1, TRPM8.)^9^. However in our model of human monocyte-derived macrophages we did not observed TRPM8 and TRPA1 gene expression. Regarding TRPV1 gene expression, its level is the same in non-CF and CF macrophages. Finally, TRPV4 is lesser expressed in CF *vs* non-CF macrophage. However, a potential agonistic effect of cannabidiol on TRPV4 activity is controversial^33–35^. Thus, we focused our study on the involvement of TRPV2 in macrophage phagocytosis capacity using the most specific TRPV2 inhibitor known, tranilast^9,21–23^.

In this study, we demonstrate that, in human macrophages, TRPV2 is activated during *P*. *aeruginosa* infection. *P*. *aeruginosa*, as cannabidiol, induces a calcium signal that is totally inhibited by tranilast, which confirms that *P*. *aeruginosa*-induced calcium influx results from TRPV2 activation. Besides, ruthenium red, a broad TRPV inhibitor^9^, and tranilast decreased the phagocytosis capacity of human primary macrophages similarly to cytochalasin D, an inhibitor of actin polymerization. Therefore, in human primary macrophages, TRPV2 channel appears to be crucial for phagocytosis of the gram-negative bacteria *P*. *aeruginosa*.

Previous studies have shown that TRPV2 is located in intracytoplasmic vesicle and then recruited to the plasma membrane, especially in phagosome after stimulation^7,10^. Moreover it has been shown that Fcγ receptor must be clustered to induce signaling and increase the intracellular Ca^2+^ concentration, these processes ultimately leading to the phagocytosis and killing of microbial pathogens^4^. TRP channels located into lipid rafts lose their activity when the lipid rafts are modulated^11,12^ and lipid rafts have been demonstrated to be crucial for phagocytosis^36^. In this context, we have investigated the localization of TRPV2 in macrophage plasma membrane, including lipid rafts, and demonstrated that TRPV2 is addressed to the plasma membrane and enriched in lipid rafts after *P*.*aeruginosa* infection. So, our data are consistent with the hypothesis that the macrophage response to *P*.*aeruginosa* involves the recruitment of TRPV2 activity through plasma membrane targeting. From 30 min, TRPV2 is recruited to plasma membrane and then decrease after 60 min. This decrease might be explained by the presence of TRPV2 in the phagolysosome cup after pathogen invagination^7^.

To confirm that membrane microstructure might be critical for TRPV2 activity, we have therefore studied the impact of a raft modulator on cannabidiol-stimulated TRPV2 activity. Cholesterol, known to ease lipid raft formation, significantly enhanced TRPV2 activity, thus suggesting that increasing rigid zones within the membrane induced the calcium influx related to TRPV2 activation. Taken together these results demonstrate, that phagocytosis of *P*. *aeruginosa* is dependent on TRPV2-mediated calcium influx which is itself dependent on TRPV2 recruitment into plasma membrane lipid rafts. Recently, several studies showed that specific features of macrophage activation in CF patients play an important role during the CF disease process. In particular, an impairment in intracellular *P*. *aeruginosa* phagocytosis was described in human macrophages^18^. These data provided clear evidences that dysfunctional CFTR affects bacterial phagocytosis in macrophages. Nevertheless, the mechanisms responsible for alterations of CF macrophage phagocytosis capacity are not clearly understood, particularly during the first step when the phagocytic cup is formed. Our results showing a central role of TRPV2 in human macrophage phagocytosis led us to consider that TRPV2 function might be altered in CF macrophages and result to impaired phagocytosis. Indeed, TRPV2 gene and protein expression as well as its activity are decreased in CF macrophages, thus suggesting that TRPV2 could be targeted to restore phagocytosis capacity. This might have therapeutics applications notably regarding *P*. *aeruginosa* infection that is responsible for chronic infection and the resulting destruction of the lungs in adult CF patients. Our data presently show that TRPV2 activity is markedly decreased although not fully abrogated. As we have demonstrated that promoting lipid raft formation increased TRPV2 activity, we proposed that the membrane physico-chemical alteration in CF macrophages could prevent the recruitment and preferential localization of TRPV2 into lipid rafts thereby limiting TRPV2-dependent phagocytosis. Moreover, it has been previously demonstrated that PI3K promotes TRPV2 recruitment to the membrane^37^. Yet, the PI3K/Akt pathway was shown to be blunted in CF pathology^38^ and this could disrupt TRPV2 trafficking to the plasma membrane in CF macrophages. However, if TRPV2 is present at the plasma membrane, no information allows us to clarify whether TRPV2 is located in the fluid area or cluster after *P*. *aeruginosa* infection. Although the link between plasma membrane alteration of CF patients and TRPV2 activity is not resolved, a therapeutic strategy for increasing TRPV2 activity could be considered, in the same way that it could be done to restore CFTR activity with ivacaftor^39^. In fact, like TRPV2 in macrophages, CFTR was shown to be recruited into lipid rafts at the plasma membrane of lung epithelial cells^40–42^. However, inhibition of CFTR function with CFTR_inh172_ did not influenced expression of TRPV2 whereas its activity was strongly decreased. The direct effect of CFTR inhibition on Ca^2+^ channels activity and on TRPV2 recruitment is poorly studied. In non-CF cells, there is no evidence in favor of the involvement of apical CFTR activity in the regulation of Ca^2+^ homeostasis. It was demonstrated in CF epithelial cells that calcium signaling is regulated by CFTR_inh172_, which suppresses Ca^2+^ release from intracellular stores^43^ and reduces Ca^2+^ influx through its modulation of store-operated Ca^2+^ channels^44^. Finally, experiments using CFTR_inh172_ showed that the presence but not the channel activity of F508del-CFTR at the plasma membrane is required to decrease the Ca^2+^ mobilization in CF epithelial cells where the abnormal trafficking of F508del-CFTR proteins were corrected^43^. So the regulation of calcium homeostasis seems to be different in CF and non-CF cells. In CF cells this seems to be dependent on the presence of CFTR at the plasma membrane whereas in non-CF cells we contribute to show that CFTR activity influences calcium homeostasis by demonstrating inhibition of TRPV2 activity by CFTR_inh172_. Among others, one possible hypothesis is that TRP channels constitute a missing link between the abnormal Ca^2+^ levels observed in CF cells and CFTR dysfunction. Indeed, TRPC4 may interact more directly with CFTR by forming a signaling complex *via* binding of their PDZ-binding motifs to PDZ-domain proteins^45^. It is therefore tempting to speculate that TRPs might be either regulators of CFTR or targets of CFTR regulating proteins. Moreover, recent evidence also suggests that both wt-CFTR and F508del-CFTR plasma membrane proteins down-regulate the TRPC6-mediated Ca^2+^ influx and TRPC6 up-regulates CFTR-dependent Cl^−^ transport^46^. However, it is not reported in the literature that the chloride imbalance observed in CF could have an impact on TRPV2 expression, on the plasma membrane or on lipid rafts.

In conclusion, CF macrophages exhibit a defect in TRPV2-mediated calcium influx and an alteration of membrane integrity that could partially explain their inability to perform efficient phagocytosis and bacterial clearance. Therefore, TRPV2 might be considered as a new target to restore phagocytosis capacity of CF macrophage and to increase innate immune defense of patients with CF.

## Methods

### CF patients

The experiments were conducted according to the Good Clinical Practice guidelines (Kong, 1997) and approved by the Ethical Committee of human subjects of the Rennes University Hospital (France, Ethics No. 11/38–827). All patients included in this study gave their written informed consent. 22 adult patients with CF were recruited at the ‘*Centre de Ressources et de Compétences de la Mucoviscidose*’ of the Rennes University Hospital (France). CF patients considered for inclusion were Caucasian and included 11 males and 11 females, who were aged between 18–58 years (mean age: 31 ± 9). The CF diagnosis was based on typical clinical manifestations of the disease and confirmed by positive sweat tests and by CFTR gene mutation detection. Stable patients were defined by the absence of changes in symptoms during the 3 months prior to the study. Patient characteristics were in the supplementary Table S1. Patients with the G551D or F508del mutation were not treated with ivacafor and/or lumicaftor at the time of their participation in the study (except for patient 18, supplementary Table S1). Blood monocyte counts were within the normal range, with a median number of 0.657 × 10^9^/L (range 0.41–0.93 × 10^9^/L).

### Cell culture

Leukocytes were isolated by Ficoll gradient centrifugation, as described previously^47^. Peripheral blood mononuclear cells from healthy non-CF subjects (written consent for the use of blood samples for the research protocol was obtained, according to the regulation for blood transfusion of the French blood organization EFS, Rennes) were seeded according to the specific blood count of each subject. Monocytes, which were selected *via* a 1-hour adhesion step, were differentiated in macrophages for 6 days using GM-CSF (400 UI/ml) in RPMI 1640 medium supplemented with 2 mM glutamine, 10% heat-inactivated fetal calf serum, 100 IU/ml penicillin and 100 μg/ml streptomycin. For phagocytosis assay, monocytes were selected using anti-CD14 beads (Miltenyi Biotec). Cell treatments were in the supplementary data.

### Calcium measurements

Intracellular Ca^2+^ concentration changes were measured using the fluorescent probe Fura-2 AM (Molecular Probes). Macrophages were incubated with 5 μM of Fura-2 AM for 45 min in the dark at 37 °C. They were washed twice with Hank’s Balanced Salt Solution (HBSS) buffer (143 mM NaCl, 5.6 mM KCl, 0.34 mM, Na_2_HPO_4_, 0.44 mM KH_2_PO_4_, 0.42 mM NaHCO_3_, 5.6 mM glucose, 10 mM HEPES, 2 mM CaCl_2_, 0.8 mM MgCl_2_ adjusted to pH 7.2). Then coverslips were mounted in observation chambers. Ratiometric calcium imaging was performed with an inverted DMIRB fluorescence microscope. Cells were excited at 340 and 380 nm using a lambda DG4 rapid wavelength switching system and acquired at 510 nm using a coolsnap HQ CCD camera. Paired images were collected every 10–30 sec during 20 min and analyzed with Metafluor software. Basal fluorescent level is recorded for 100 sec, afterwards the coverslips is available for treatments. At the end of the experiment, ionomycine (5 µM) was used as positive control. Fluorescence changes were expressed as the ratio F340/F380 normalized to basal value 1. Moreover, for CF macrophages experiments, maximal level obtained by ionomycin were normalized to value 4 (mean maximal level in CF macrophages). Area under curve (AUC) is determined between signal and basal line (Y = 1) after 100 sec. At 100 sec, treatment was added and AUC was measured between 100 sec until 1250 sec. Average AUC from multiple independent coverslips were used to calculate the sample mean ± s.e.m obtained after analysis of at least 10 cells.

### Phagocytosis assay

Phagocytosis capacity was evaluated using heat-inactivated *E*. *coli* linked to fluorescein by means of the ‘Vybrant Phagocytosis Assay kit’ (Molecular Probes, ThermoFisher Scientific).

### Biotinylation assay

TRPV2 channel membrane expression was assessed by membrane surface biotinylation assay. Macrophages were treated with *P*. *aeruginosa* (MOI 50) for 5, 10, 30 or 60 min. After incubation, cells were washed twice with biotinylation buffer (PBS supplemented with 0.1 mM CaCl_2_ and 1 mM MgCl_2_) and incubated for 30 min at 37 °C with EZ-link sulfo-NHS-LC-Biotin (0.5 mg/ml, ThermoFisher Scientific). Then the reaction was quenched by bovine serum albumin (BSA 0.1%) and cells were washed with PBS. Next, cells were lysed in radio-immunoprecipitation assay (RIPA) buffer (50 mM Tris-HCl pH 7.5, 150 mM NaCl, 25 mM NH_4_Cl 1 mM EDTA, 0.1% SDS, 1% Triton-X100, 12 mM deoxycholate, 2 mM NaF, 1 mM Na_3_VO_4_, 2 mM PMSF, cOmplete™ EDTA-free Protease Inhibitor Cocktail and phosphatase inhibitor cocktail 2), and incubated at 4 °C for 30 min in lysis buffer, with vortexing for 30 sec every 10 min. The supernatant that contain proteins was obtained by subsequent centrifugation at 10,000 g for 10 min at 4 °C, and the protein concentration was determined using BCA protein assay. To capture biotinylated proteins, 100 µg of total proteins in 0.5 ml of RIPA buffer were incubated overnight at 4 °C under rotation with 50 µl of high capacity streptavidin agarose resin (ThermoFisher Scientific). Following this incubation, membrane proteins were washed three times, 5 min at 4 °C under rotation, with lysis buffer and pull-down between steps. Captured proteins were eluted in Laemmli 2X buffer, boiled at 95 °C for 5 min, and analyzed by immunoblot as described below.

### Immunoblotting

Total proteins were extracted from macrophages by lysing cells with RIPA lysis buffer. The protein concentration was determined using a BCA protein assay kit. Proteins were separated via SDS-PAGE and transferred to a nitrocellulose membrane. Then, membrane was subjected to western blotting using a rabbit anti-TRPV2 antibody, a mouse anti-HSC-70 antibody (anti-VRL-1 (vanilloid receptor-like 1) antibody, Santa Cruz), a rabbit anti-CD71 antibody (Cell Signaling Technology) or a mouse anti-flotillin-1 antibody (BD bioscience). Horseradish peroxidase-conjugated goat anti-rabbit (Cell Signaling Technology) or goat anti-mouse (Dako) antibodies were used as secondary antibodies, and proteins were detected using enhanced chemiluminescence. The images were scanned with the Fujifilm LAS-3000 imager and analyzed with the MultiGauge software for densitometry. The intensity of the bands was normalized relative to HSC-70.

### Immunofluorescence staining

Following treatment, macrophages were fixed using PFA 4%-sucrose 4% in PBS for 15 min at 4 °C. Then quenching of fluorescence was realized with glycine (0.1 M) for 30 min and cells were blocked for 30 min in PBS-1% FCS-0.2% saponine. Afterwards, they were stained using the blocking solution containing rabbit anti-TRPV2 antibody (anti-VRL-1 (vanilloid receptor-like 1) antibody, Santa Cruz) for 20 min at 4 °C, followed by incubation with goat anti rabbit secondary antibody coupled to green fluorescent Alexa Fluor 488 (Molecular Probes, ThermoFisher Scientific) for an additional 20 min. Finally, cells were washed three times and co-stained by 10 min-incubation in a blocking solution containing Hoechst to dye nuclei and WGA to dye membrane. After washing, coverslips were mounted with mounting solution. Fluorescent-labeled cells were captured using a confocal Leica SP8 microscope with the Leica LASAF software. When lipids rafts were analyzed, coverslips were stained using a blocking solution (PBS-1% FCS) containing cholera toxin subunit B (CTX-B) coupled to red-fluorescent Alexa Fluor 647 (Molecular Probes, Life Technologies) for 30 min at 4 °C before fixation. Fluorescence quantification and co-staining was examined using FIJI software. For fluorescence quantification, data were obtained after passed the images in a macro. The TRPV2 membrane versus cytosolic fluorescence intensity was obtained after particles identification using the control WGA (wheat germ agglutinin) fluorescence (Supplementary Fig. S5) as describe below. The threshold of the WGA image was fix to 3, 255. Then the image was converted to mask, the holes were filled and erode and dilate to erase background. These first mask corresponding to whole cells was duplicated and saved. Using this first mask, ten steps of dilatation were effected defining the cytoplasmic part of the cells. This second mask was also duplicated and saved. The second mask was inverted and multiply with the first mask to obtain a third mask representing only the membrane part. These two last mask, cytoplasmic and membrane, were used on TRPV2 images to quantify separately the intensity of the cytoplasmic and membrane parts. The GM1 fluorescence intensity was only quantified on membrane part of the cells. Observations were realized on at least three different fields of coverslips.

### Lipid raft isolation by lysis gradient centrifugation

Lysis gradient centrifugation is performed as described by Schatzlmaier *et al*.^48^. Briefly, 3 × 10^6^ primary human macrophages were suspended in 1 ml ice cold PBS-crystal violet (5 µg/ml) and overloaded on a gradient prepared with discontinuous iodixanol concentration fraction on ice (35%, 20%, 10% and 5%; down to up). The 10% iodixanol fraction (lysis layer) contains 0.5% detergent NP-40 (ThermoFisher Scientific), cOmplete™ EDTA-free Protease Inhibitor Cocktail and phosphatase inhibitor cocktail 2 (Sigma Aldrich). Following centrifugation for 10 min at 1000 g, 200 µl of the C + DSM (Cytoplasm + Detergent Soluble Membrane), up to the 10% fraction, and of the N + DRM (Nuclei + Detergent Resistant Membrane), up to the 35% fraction, were harvested. A volume of 25 µl of each fraction was set aside for cholesterol level quantification. Universal nuclease (ThermoFisher Scientific) was added to the fraction, incubated for 30 min and sonicated for immunoblotting. The protein concentration was determined using a BCA protein assay kit. Quantitative separation was controlled after equal loading of protein on SDS-PAGE. For TRPV2 analysis, C + DSM and N + DRM (1:3; w/w) proteins were separated *via* SDS-PAGE, as described previously.

### Gene expression

RNA expression was analyzed using RT-qPCR assays as previously described^49^. The gene-specific primers for 18 S (Forward: CGCCGCTAGAGGTGAAATTC; Reverse: TTGGCAAATGCTTTCGCTC) and TRPV2 (Forward: AGCATCTGGAAGCTGCAGAAAG; Reverse: TGGGCCATCAGTTGGACTGG), TRPA1 (Forward: TGCATGTTGCATTCCACAGAAG; Reverse: TTGAGGGCTGTAAGCGGTTCATA), TRPM8 (Forward: CACCTCAGAGGAAATGAGGCAT; Reverse: GCAATCTCTTTCAGAAGACCCTTG), TRPV1 (Forward: GACCTGTGCCGTTTCA; Reverse: CCTGTGCGACGTGGACTCA) and TRPV4 (Forward: CTACGCTTCAGCCCTGGTCTC; Reverse: GCAGTTGGTCTGGTCCTCATTG) were purchased from Eurogentec. The amplification curves of the PCR products were analyzed with the ABI Prism SDS software using the comparative cycle threshold (CT) method. Relative gene expression was calculated by comparing the number of thermal cycles that were necessary to generate threshold amounts of product (CT). The CT was calculated for each gene and for the housekeeping gene 18 S. For each cDNA sample, the 18 S CT was subtracted from the CT for each gene to yield the ΔCT, thus normalizing the initial amount of RNA used. The amount of mRNA was calculated as 2^–ΔΔCT^, where the ΔΔCT is the difference between the ΔCT of the two cDNA samples to be compared. The data from CF samples are expressed relative to the mRNA level found in non-CF samples.

### Statistical analysis

The number of subjects and experiments used in each group is quoted in figures. Statistical significance was evaluated by using the GraphPad Prism software v.7.0 (GraphPad Software Inc., San Diego, CA, US). A non-parametric Mann-Whitney test was used to assess the statistical significance of differences. For each analysis, a p-value < 0.05 was considered to be significant.

### Data availability statement

Supporting data are available to editors and peer reviewers for the purposes of evaluating the manuscript.

## Electronic supplementary material

supplementary data set 1
