# Supplementary material for: Phagocytosis depends on TRPV2-mediated calcium influx and requires TRPV2 in lipids rafts: alteration in macrophages from patients with cystic fibrosis
Source: Sci Rep. 2018 Mar 9;8:4310. doi: 10.1038/s41598-018-22558-5 (PMC5844937; doi:10.1038/s41598-018-22558-5)

**Description of supporting information**

**Phagocytosis depends on TRPV2-mediated calcium influx and requires TRPV2 in lipids rafts: alteration in macrophages from patients with cystic fibrosis.**

Manuella Lévêque^1,2^, Aubin Penna^1,2^, Sophie Le Trionnaire^1,2^, Chantal Belleguic^3^, Benoît Desrues^3,5^, Graziella Brinchault^3^, Stéphane Jouneau^3,4^, Dominique Lagadic-Gossmann^1,2^ and Corinne Martin-Chouly^1,2,*^

**SUPPORTING INFORMATION METHODS**

**Cell culture and treatments**

Before treatment, the macrophages were placed overnight in fresh medium supplemented with 1.5% of FCS. To study TRPV2 activity, primary human macrophages were stimulated using *P. aeruginosa* (MOI 50, strain PaO1) or *E. coli* (K-12 strain) BioParticles® conjugated to fluorescein. Cells were treated or not by a TRPV2 specific inhibitor, the tranilast (100µM), for 15 min before experiments or ruthenium red (20µM) and during phagocytosis capacity assay or calcium measurement. Recruitment of TRPV2 in plasma membrane, especially in lipids rafts, were studied in primary human macrophages treated or not by *P. aeruginosa* for the time indicated in figures. To increase the rigidity of the lipid rafts, cells were treated by water-soluble cholesterol (15µg/ml) for 30 min before experiment and during calcium measurement. Inhibition of the CFTR function were studied by treatment of human macrophages with CFTR_inh-172_ (10µM; added every 24h during 72h). During calcium measurements, cells were stimulated by cannabidiol (75µM), a pharmacological activator of TRPV2. At the doses used, the treatments did not affect the viability of macrophages (data not shown). Viability was measured using the CellTiter 96® AQueous One Solution Cell Proliferation Assay (Promega) according to manufacturer’s instructions.

**SUPPORTING INFORMATION FIGURE LEGENDS**

**Figure S1. Heat inactivated *E. coli*** **induced TRPV2 mediated calcium influx in primary human macrophage.** TRPV2 mediated-Ca^2+^ influx in macrophages stimulated by *E. coli* (K-12 strain) (MOI 50) in the absence (control) and presence of tranilast (100µM). Data are presented as the ratio of emission after excitation at 340 nm to that after excitation at 380 nm (F340/F380) normalized to basal level 1. Horizontal bar represented stimulus period. Data are representative of three independent experiments. Below, area under curve of the same experiments are shown as mean ± s.e.m. Mann-Whitney test: *p<0.05 *vs.* control.

**Figure S2. (*A*) LPS from *P. aeruginosa*-induced TRPV2 recruitment into lipid rafts from human macrophage membrane.** Macrophages treated by LPS from *P. aeruginosa* were analyzed by quantitative separation of C+DSM (cytoplasm and detergent-soluble membrane) and N+DRM (nuclei and detergent-resistant membranes) fractions using lysis gradient centrifugation. Both fractions were resolved on reducing gel and analyzed by immunoblotting. Representative blots of four separate experiments are shown. CD71 and flotillin-1 are cell compartment markers for C+DSM and N+DRM respectively. Below densitometric analysis of TRPV2 in the N+DRM fraction of the same experiments are shown as mean ± s.e.m. The results were expressed *vs.* the relative intensity observed in control. Mann Whitney test: *p<0.05 vs control. **(*B*) Cholesterol level into DRM and DSM fractions.** Human macrophage were infected or not (control) by *P. aeruginosa* (MOI 50, 60min). Then cell lysates were analyzed by quantitative separation of C+DSM (cytoplasm and detergent-soluble membrane) and N+DRM (nuclei and detergent-resistant membranes) fractions using lysis gradient centrifugation. Total cholesterol levels were determined in the C+DSM and N+DRM fractions using Cholesterol/Cholesteryl Ester Quantitation Kit. Data are shown as mean ± s.e.m. The results were expressed *vs.* the relative intensity observed in control (n=5). Mann Whitney test: *p<0.05 *vs*. C + DSM untreated and ##p<0.01 *vs*. C + DSM treated with *P. aeruginosa* (MOI 50).

**Figure S3: TRPV1 and TRPV4 gene expression in non-CF and CF macrophage.** TRPV1 (A) and TRPV4 (B) mRNA expression were determined by RT-qPCR in non-CF and CF macrophages (n=6). Mann-Whitney test: * p<0.05 *vs.* non-CF macrophages.

**Figure S4: Full-length blots in the main article.** Full-length blots in the main article from Figure 3B (*A*), Figure 4B (*B*), Figure 6B (*C*) and Figure 7B (*D*). Red dotted lines show the cropping locations.

**Figure S5: Confocal pictures of WGA** **in the main article from Figure 3A.** Confocal pictures of WGA localization in human primary macrophages membrane after *P. aeruginosa* treatment (MOI 50, time 0 to 60min). The yellow line define the cell outline obtain after passing the images in macro. Images are representative of four separate experiments.

**SUPPORTING INFORMATION DATA AND FIGURES**

**Table S1:** Characteristics of CF patients

**Abbreviations :** A.f.: *Aspergillus fumigatus;* P.a.: *Pseudomonas aeruginosa*; S.a.: Staphylococcus *aureus*

| **Patient** | **Age** | **Sex** | **Genotype** | **Microbiology** | **BMI** | **FEV1 % predicted** |
| --- | --- | --- | --- | --- | --- | --- |
| **1** | 27 | F | F508del / F508del | A.f / S.a | 20,5 | 97,2 |
| **2** | 35 | F | F508del / F508del | P.a / S.a | 19,1 | 30,2 |
| **3** | 28 | F | 1248+1G→A | A.f / S.a | 20,7 | 90,5 |
| **4** | 40 | M | F508del / F508del | P.a / A.f | 21,9 | 48,7 |
| **5** | 29 | M | F508del / F508del | A.f / S.a | 19,58 | 69,5 |
| **6** | 18 | F | F508del / F508del | None | 24,1 | 100,4 |
| **7** | 20 | F | F508del / S945L | A.f / S.a | 21,3 | 68,4 |
| **8** | 24 | F | F508del / F508del | P.a / A.f / S.a | 16,5 | 28,4 |
| **9** | 26 | F | F508del / F508del | P.a / A.f / S.a | 19,09 | 67 |
| **10** | 41 | F | F508del / R117H | None | 22,52 | 92,6 |
| **11** | 39 | F | F508del / F508del | - | 19,06 | 71,5 |
| **12** | 30 | M | F508del / F508del | A.f/S.a | 19,9 | 65,9 |
| **13** | 26 | M | F 311L / N1303K | S.a | 18,38 | 94,1 |
| **14** | 31 | M | F508del / G91R | A.f/S.a | 24,42 | 73,4 |
| **15** | 27 | M | F508del / F508del | S.a | 24,88 | 61 |
| **16** | 25 | M | F 311L / N1303K | S.a | 18,45 | 91,4 |
| **17** | 22 | M | F508del / 4016insT | S.a | 17,75 | 73,9 |
| **18** | 45 | M | F508del / G551D | S.a | 22,22 | 25,8 |
| **19** | 30 | F | F508del / G551D | A.f/S.a | 22,86 | 104,5 |
| **20** | 58 | M | F508del / 2789+5G→A | P.a/ S.a | 25,34 | 90,1 |
| **21** | 23 | M | W119X / G551D | None | 23,5 | 109 |
| **22** | 29 | F | 1248+1G→A | P.a / A.f | 21,5 | 110,6 |

*FEV1: Force Expiratory Volume in one second;* (-) : no data


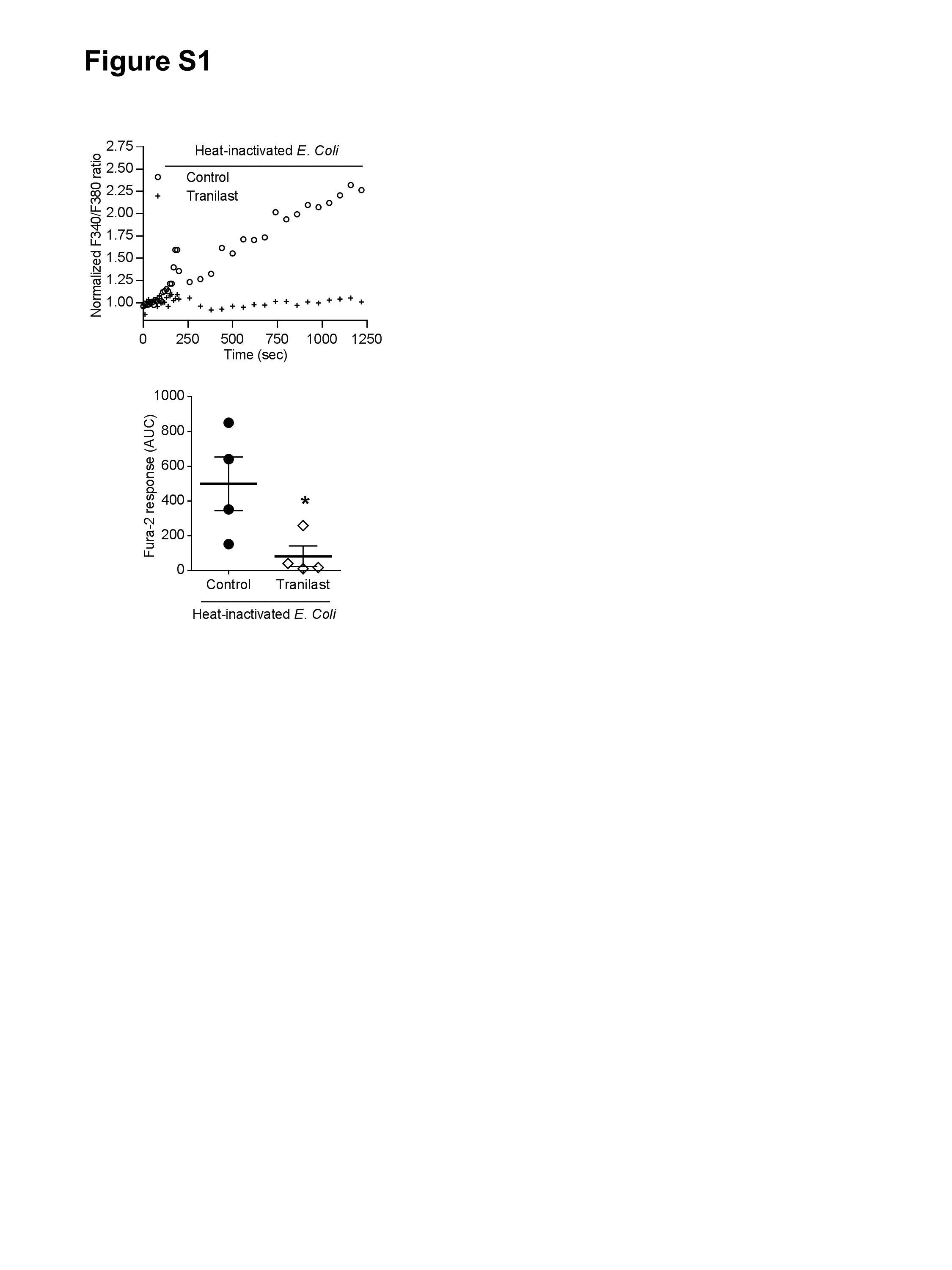


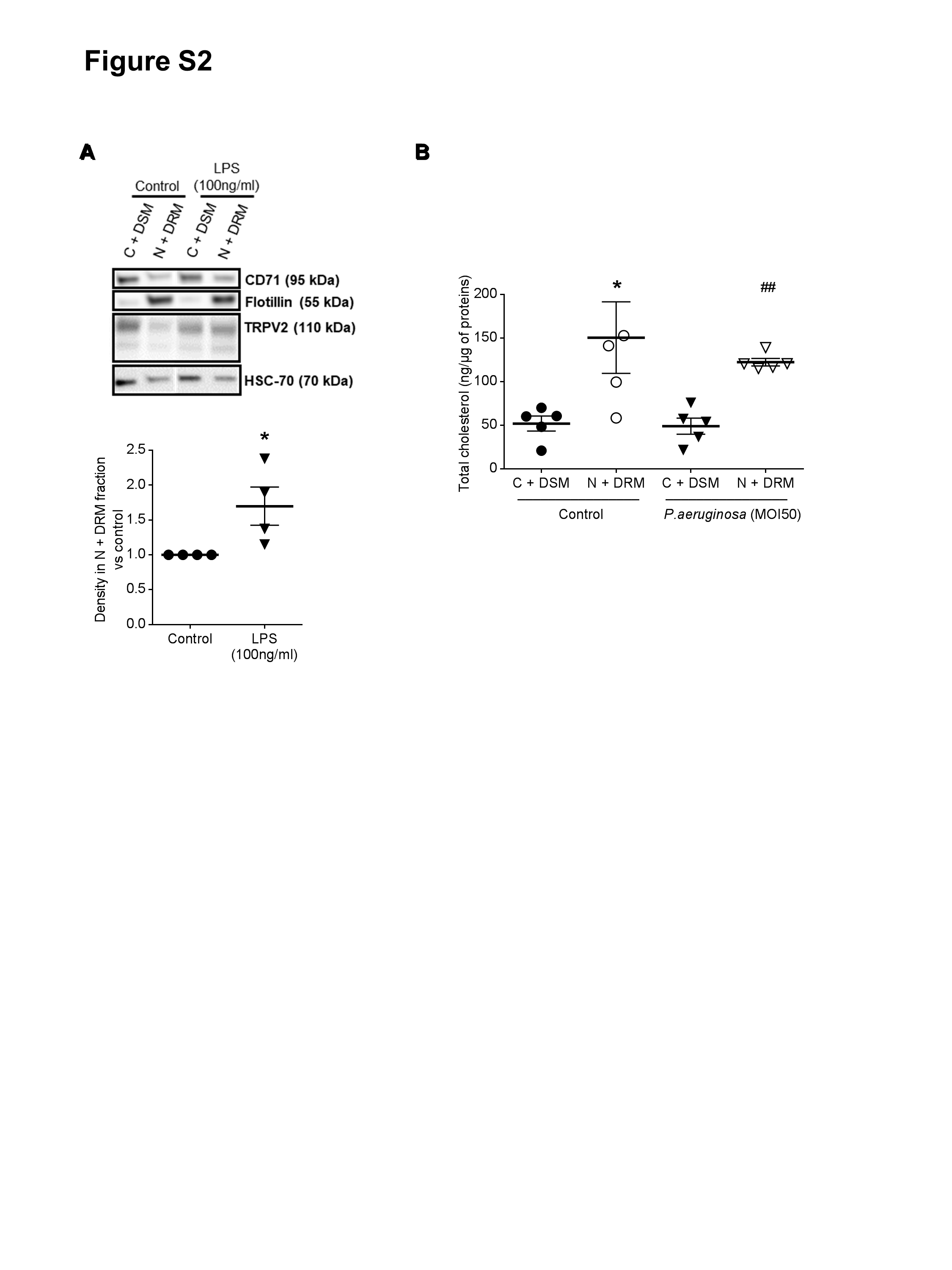


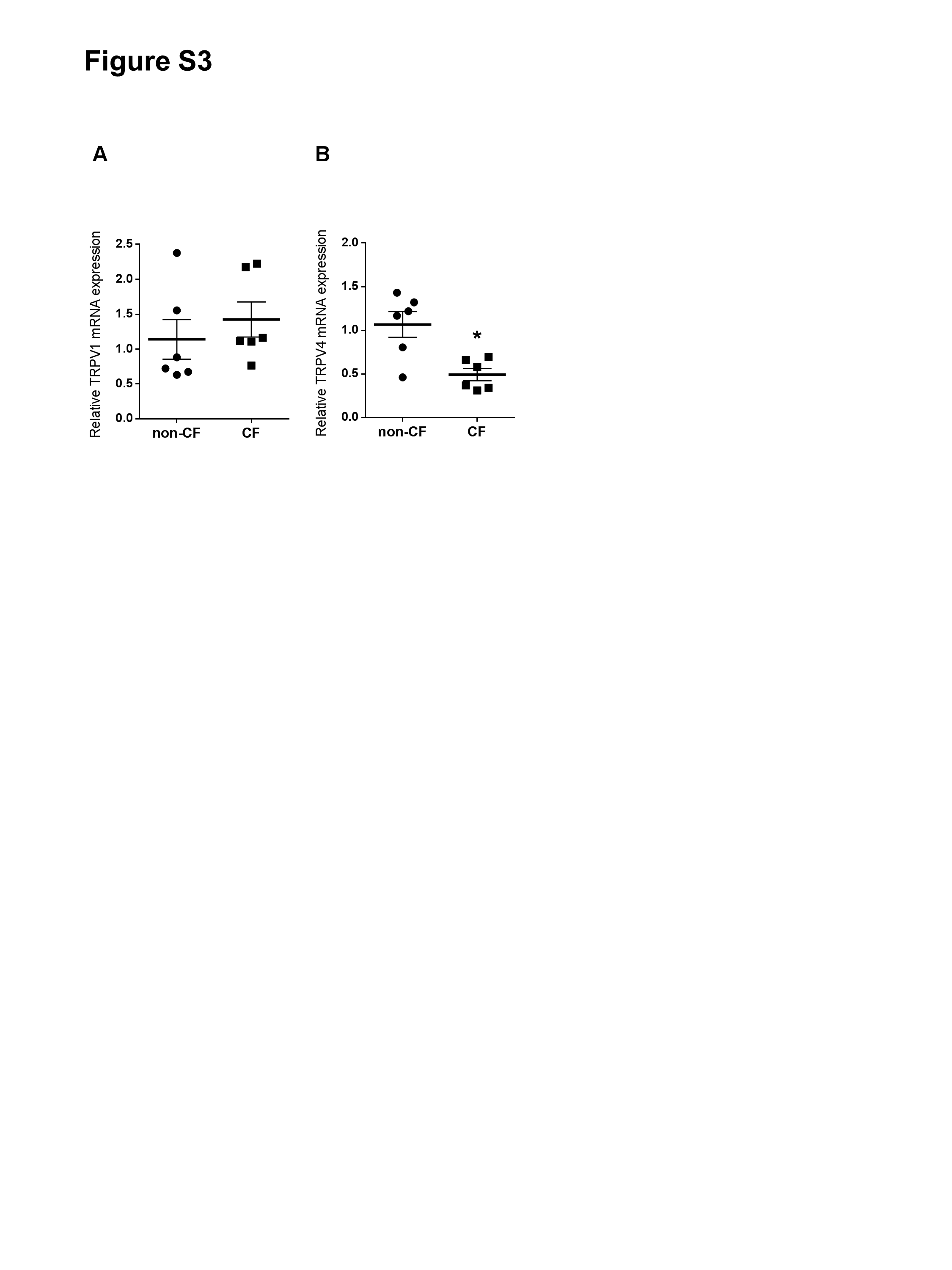


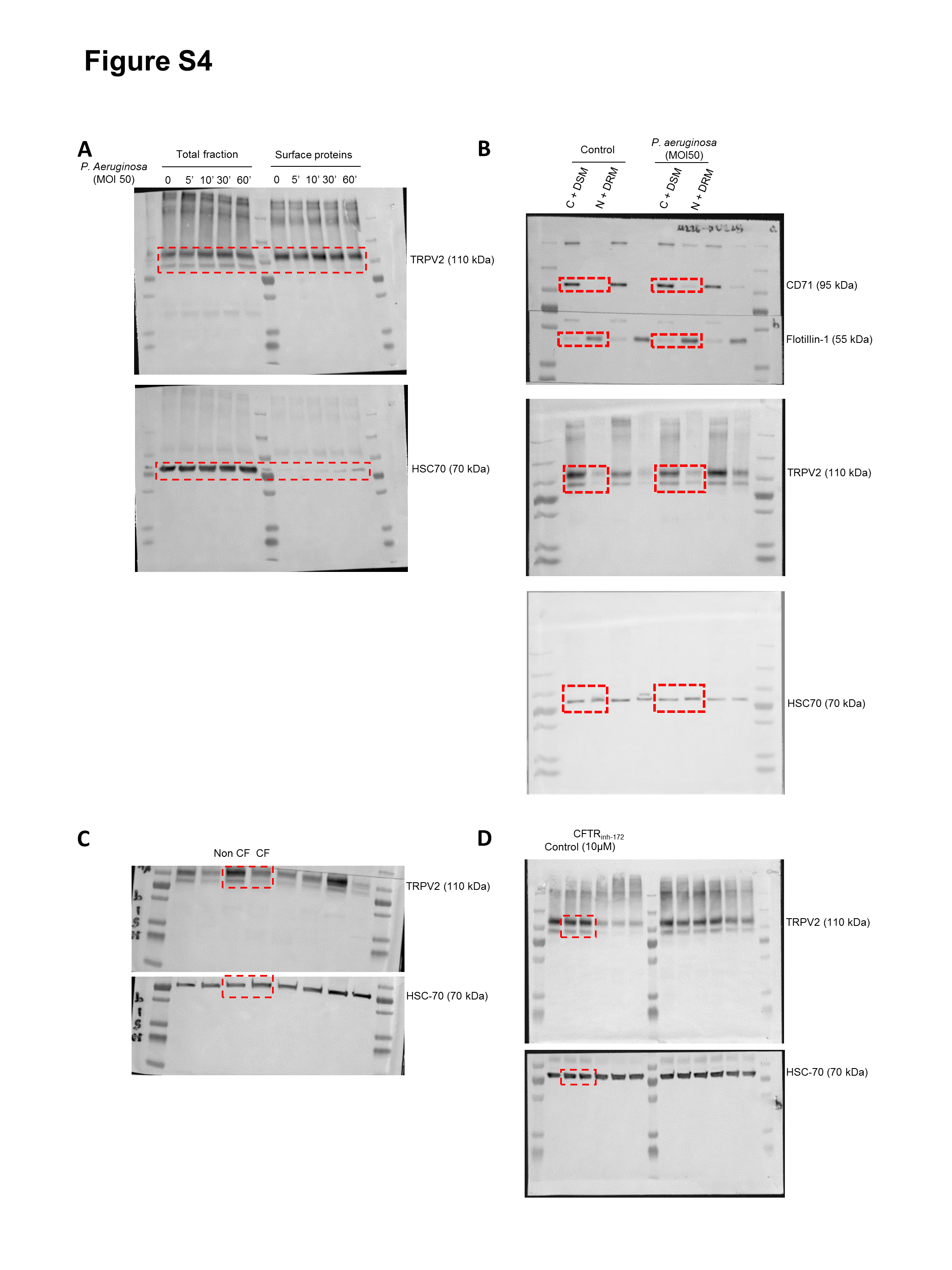


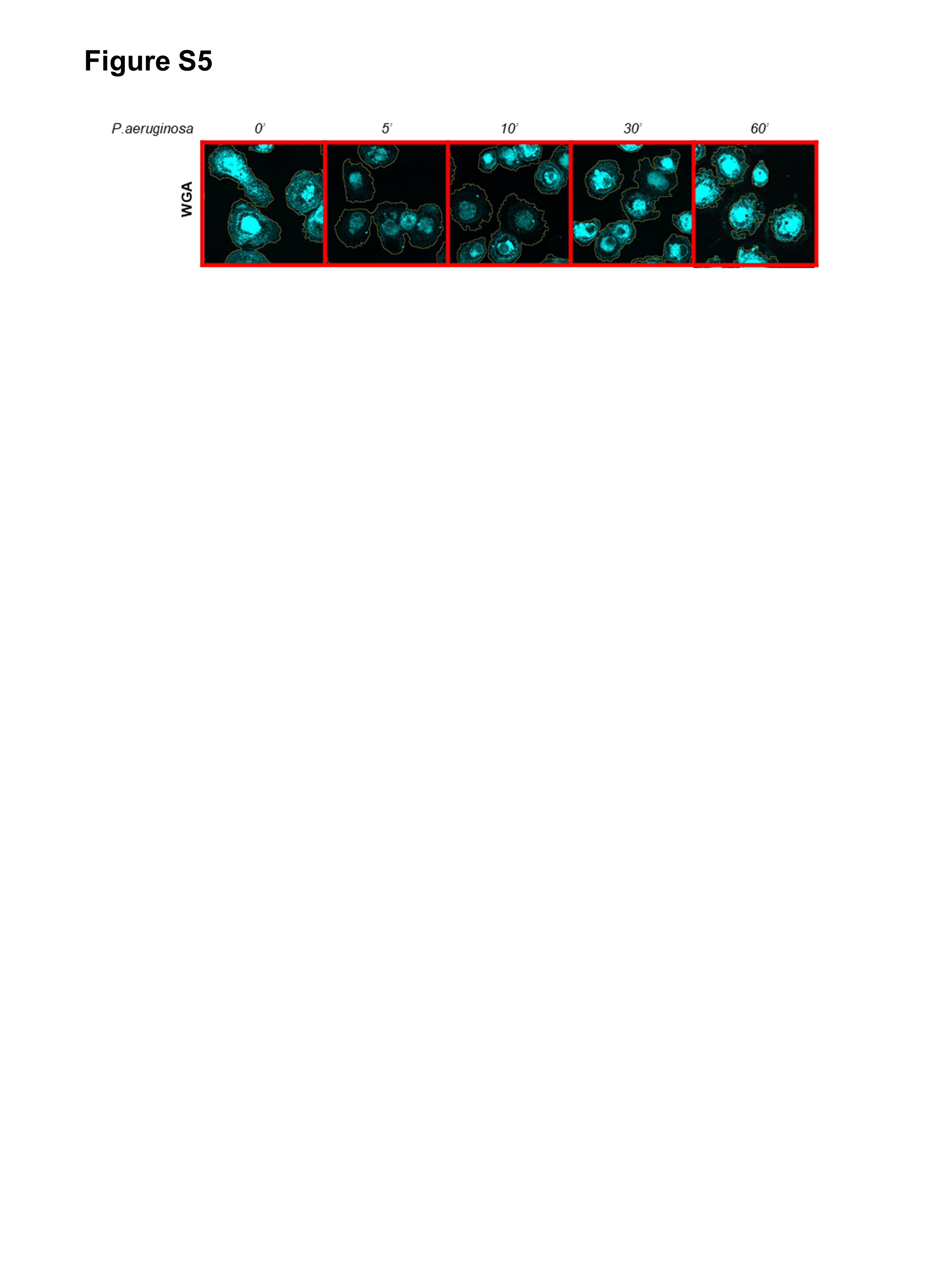

Supplement: Supplementary file 1 — supplementary data set 1 [file 41598_2018_22558_MOESM1_ESM.doc]
